# Initiator enhancement of mandrel degradation for ICF target fabrication

**DOI:** 10.1016/j.isci.2022.104733

**Published:** 2022-07-09

**Authors:** Qiang Chen, Yu Zhu, Zhanwen Zhang, Jiajun Ma, Zhibing He, Zhigang Wang

**Affiliations:** 1 Laser Fusion Research Center, China Academy of Engineering Physics, Mianyang 621900, China; 2 Institute of Atomic and Molecular Physics, Jilin University, Changchun 130012, China; 3 State Key Laboratory of Environmental-friendly Energy Materials, School of Material Science and Engineering Southwest University of Science and Technology, Mianyang 621010, China

**Keywords:** Atomic interactions, Nuclear physics, Plasma confinement

## Abstract

Poly-α-methylstyrene (PAMS), as an ideal mandrel material used in the fabrication of inertial confinement fusion (ICF) targets, its efficient degradation is the key to the quality of targets. However, there is a great challenge to achieve enhanced degradation. Here, we proposed the strategy to optimize the degradation of PAMS microspheres using di-*tert*-butyl peroxide (DTBP) as a degradation initiator. Experimentally, monodisperse PAMS microspheres with DTBP were controllably prepared by a microfluidic-based microencapsulation technique. Thermogravimetric results show that DTBP largely decreases the initial degradation temperature from 550 K to 450 K, which effectively promotes the thermal degradation of PAMS microspheres. Theoretically, DTBP can reduce the activation energy of degradation. Moreover, the potential energy surfaces were used to describe the degradation process at the atomic level. Our work brings a new direction for the study of mandrel degradation in ICF targets fabrication, and also provides a valuable reference for solving the pollution of waste plastics.

## Highlights

• Di-*tert*-butyl peroxide enhances the thermal degradation performance of mandrel
• Di-*tert*-butyl peroxide decreases the degradation activation energy of mandrel
• Thermogravimetric study and DFT calculation prove the enhanced degradation

## Graphical abstract

**Figure:**
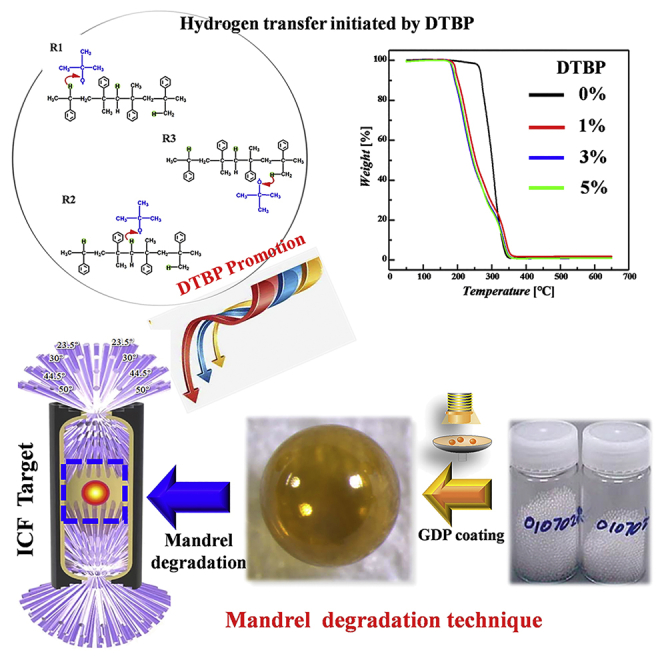

## Introduction

The polymer degradation is not only an important topic in the environment protection (Wang et al., 2016; Yang et al., 2009; Guo et al., 2012; Rhodes, 2018; Xu et al., 2019) but also the key process for the fabrication of nuclear fuel targets used in inertial confinement fusion (ICF) (Zhang et al., 2020; Glenzer et al., 2010; Betti and Hurricane, 2016; Hurricane et al., 2014). In particular, the latest progress in achieving burning plasma and reaching the ignition threshold signals a big step toward delivering the promise of nuclear fusion as the ultimate solution to energy problems (Kritcher et al., 2022; Zylstra et al., 2022; Clery, 2021). As the quality of ICF targets can directly influence the ignition experiments, there is an urgent need to develop new strategies to improve the targets. Generally, the mandrel degradation technique, which consists of three steps including mandrel fabrication, layer coating and degradation, is the basis of ICF targets fabrication (McQuillan et al., 1997). Specifically, poly-α-methylstyrene (PAMS) microspheres are firstly prepared and used as mandrel templates; the microspheres are then coated with glow discharge polymer (GDP) by chemical vapor deposition, forming GDP/PAMS compound shells; according to the difference in degradation temperature between the GDP layer and the PAMS microsphere, the compound shells are thermally treated to remove the inner PAMS microspheres and the hollow GDP targets are finally obtained (Nikroo and Pontelandolfo, 2000). Therefore, the degradation of PAMS microspheres is very significant to the final GDP targets. Considering that both the GDP layer and the PAMS microspheres are hydrocarbon materials, and the difference in degradation temperature is small, it is a crucial topic to achieve more effective degradation of PAMS microspheres without damaging the GDP layer.

In the past, numerous works have been conducted to investigate the thermal degradation of PAMS. The degradation model which combined depolymerization and hydrogen-transfer-induced chain scission at the atomic level has been well established (Madras et al., 1996; Madras and McCopy, 1997). Researchers believe that the degradation of PAMS is initiated by free radicals generated from random chain scission (Yu et al., 2015; Simha et al., 1950). Moreover, there is degradation uncertainty caused by quantum tunneling during thermal degradation (Zhu et al., 2022). It should be noted that both the random chain scission and degradation uncertainty would lead to the degradation of PAMS deviating from the ideal depolymerization process and leaving residues. However, the attempts on developing strategies to enhance the degradation of PAMS to protect the GDP layer and avoid residues remain lacking. As hydrogen-transfer-induced chain scission is the main process during PAMS degradation and free radicals can enhance polymer degradation by increasing the abstraction of hydrogen, an initiator which can generate free radicals would promote the continuous depolymerization of PAMS (Kim and McCoy, 2000; Sterling et al., 2001). And this has never been proposed in the degradation mandrel technique in ICF.

In the present work, a peroxide named di-*tert*-butyl peroxide (DTBP) is proposed as the degradation initiator to prepare the monodisperse PAMS microspheres with enhanced thermal degradation. Specially, the effects of DTBP on the degradation of PAMS microspheres are experimentally and theoretically investigated. Thermogravimetric experiments were employed to analyze the degradation performance. Then, potential energy surfaces (PESs) calculations and dynamic simulations were carried out to explore the altered PAMS degradation mechanism at the atomic level.

## Results and discussion

### Morphologies of solidified poly-α-methylstyrene microspheres

**Figure 1:**
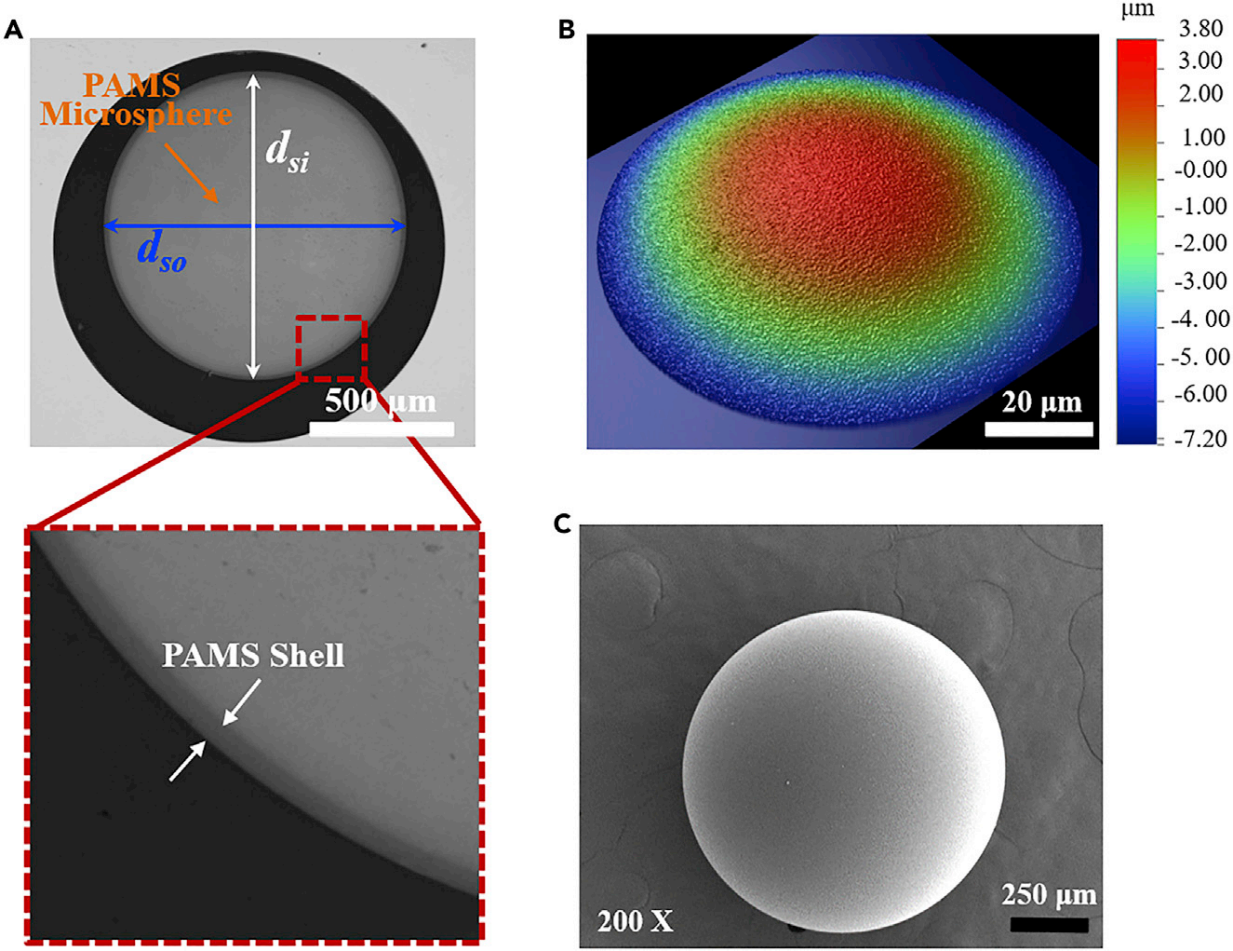
Morphologies of solidified PAMS microspheres (A) The X-ray radiography. (B) The white light interference. (C) SEM micrographs. The scale bar in the X-ray radiography photograph is 500 μm. The scale bar in the SEM micrograph is 200 μm and the magnification is 200.

The morphologies of the PAMS microspheres prepared by the microencapsulation method (Chen et al., 2019; Datta et al., 2014) are illustrated in Figure 1. As shown in Figure 1A, an X-ray radiography is employed to characterize the core-shell structure of PAMS microsphere. The inner and outer outlines of the microsphere can be clearly observed in the X-ray micrograph. Moreover, the sphericity ($\varepsilon =\left(1-\frac{d_{somax}-d_{somin}}{\bar{d_{so}}}\right)\times 100\text{\%}$), *d*_*somax*_, *d*_*somin*_ and $\bar{d_{so}}$ are the maximum diameter, minimum diameter, and mean diameter of eight outer diameters in different directions of PAMS microspheres, respectively) of the PAMS microspheres are better than 99.5%. In addition, the shell thickness of a single microsphere is also uniform. White light interference (WLI) (Figure 1B) and scanning electron microscope (SEM) (Figure 1C) are also adopted to characterize the surface morphology of PAMS microspheres (the color bar in the WLI image represents the height). From the WLI and SEM images we can see that the outer surface of the microsphere is smooth and there are no defects such as vacuole, scratches, or wrinkling onside the outer surface.

### Thermogravimetric experiments and thermal degradation kinetic

**Figure 2:**
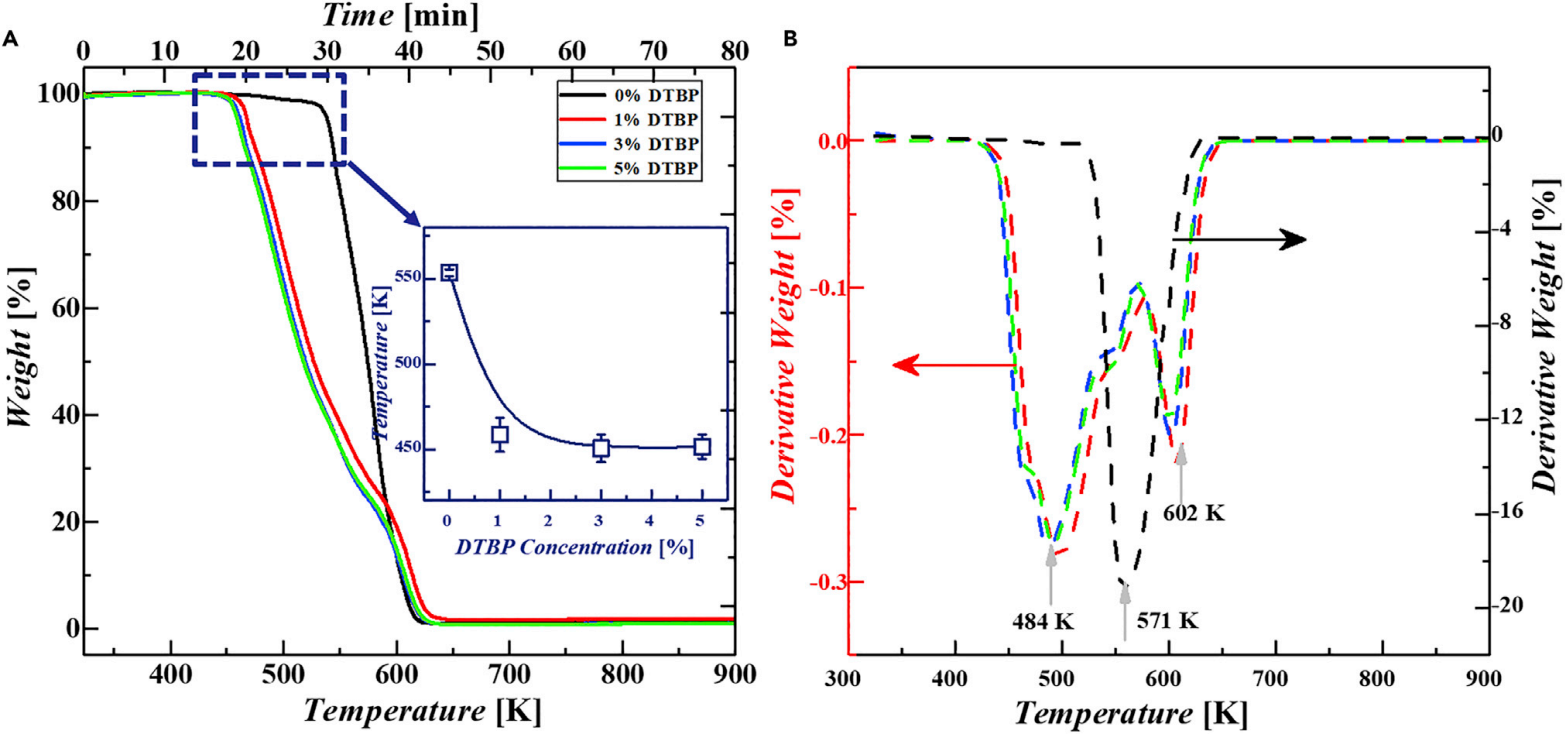
Effects of DTBP on the degradation of PAMS microspheres (A) The TG curves of the PAMS microspheres. The inset illustrates the effect of DTBP on the initial degradation temperature of PAMS microspheres. (B) The DTG curves of the PAMS microspheres. The grew arrows represent the location of peaks in the DTG curves. The black line indicates the thermal degradation of PAMS microsphere in the absence of DTBP. The multicolor lines indicate the degradation of PAMS microspheres in the presence of DTBP with various concentrations.

Thermogravimetric experiments combined with theory calculations and numerical simulations were executed to explore the effects of DTBP on the degradation performance of PAMS microspheres. In this work, the thermogravimetric (TG) and derivative thermogravimetric (DTG) curves of the PAMS microspheres prepared with various concentrations of DTBP were obtained (Figure 2). Figure 2A illustrates the effects of DTBP on the time scales and temperature of PAMS degradation. Specifically, in the absence of DTBP, PAMS begins to degrade at 550 K at the timepoint of 30th minute and fully degrades at 625 K at the time point of 40th minute. Although in the presence of DTBP, PAMS begins to degrade at 450 K at the timepoint of 20th minute and ends at 625 K at the timepoint of 40th minute. This indicates that the introduction of DTBP greatly decreases the initial degradation temperatures. The prolongation of the degradation period is beneficial to the complete reaction of PAMS. However, there is no obvious effect of DTBP concentration on the initial degradation temperature of PAMS. To discuss the effects of DTBP on the weight loss rate during degradation more clearly, the corresponding DTG curves were calculated and smoothly treated (Figure 2B). In the absence of DTBP, there is a main weight loss peak at about 571 K. In the presence of DTBP, there are two weight loss peaks in the DTG curve at about 484 K and 600 K, respectively. From Figure 2B, the weight loss rate of PAMS in the presence of DTBP is much lower than that in the absence of DTBP, which is consistent with the results of time scales shown in Figure 2A. Furthermore, from the experimental TG and DTG data, we select the weight loss peaks to analyze the effects of DTBP on PAMS thermal degradation kinetics. According to the Arrhenius temperature dependence of the reaction rate (*κ*_*i*_ = *A*_*i*_·exp(-*E*_*i*_/*RT*)), where *A*_*i*_ is the prefactor, *E*_*i*_ is the degradation reaction activation energy, *R* is the ideal gas constant and *T* is the temperature), the equation can be extended as

$$\frac{da_{i}}{dt}=A_{i}\;\exp\left(-E_{i}/RT\right)\cdot \left(1-a_{i}\right)$$

where *α*_*i*_ = (*m*_*0*_-*m*_*t*_)/*m*_0_ is the weight loss rate, *m*_*0*_ and *m*_*t*_ are the initial mass and the mass at time *t*; *κ*_*i*_ = dα_*i*_/d*t* represents the reaction rate. The following equation can be deduced by taking the logarithm and differentiating the above equation.

$$\frac{\Delta \mathrm{lg}\left(da_{i}-dt\right)}{\Delta \mathrm{lg}\left(1-a_{i}\right)}∼-\frac{E_{i}}{2.33R}\cdot \frac{\Delta \left(1/T\right)}{\Delta \mathrm{lg}\left(1-a_{i}\right)}$$

**Figure 3:**
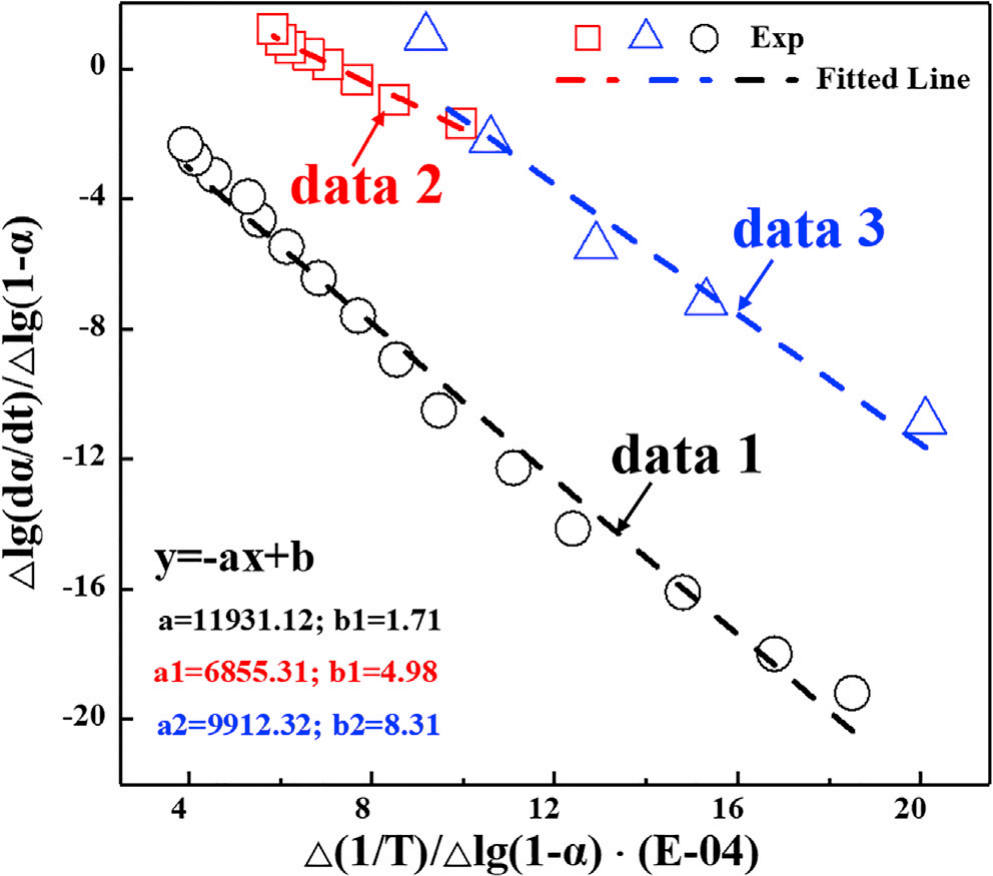
The thermal degradation kinetics of PAMS microspheres The hollow symbols represent the values obtained from the thermal degradation experiment. The dotted lines are obtained by the linear fit. The black data (data 1) represent the thermal degradation kinetics of PAMS in the absence of DTBP. The red data (data 2) represent the thermal degradation kinetics of PAMS at the first weight loss peak (T = 484 K, corresponding to Figure 2B) in the presence of DTBP. The blue data (data 3) represent the thermal degradation kinetics of PAMS at the second weight loss peak (T = 602 K, corresponding to Figure 2B) in the presence of DTBP.

With this equation, the relationship between $\frac{\text{Δlg}\left(\text{d}{\text{α}}_{i}/\text{d}t\right)}{\text{Δlg}\left(1-{\text{α}}_{i}\right)}$ and $\frac{\text{Δ}\left(1/\text{T}\right)}{\text{Δ}\mathrm{lg}\left(1-{\text{α}}_{i}\right)}\;$ can be obtained. As illustrated in Figure 3, $\frac{\text{Δlg}\left(\text{d}{\text{α}}_{i}/\text{d}t\right)}{\text{Δlg}\left(1-{\text{α}}_{i}\right)}$ is linearly dependent on $\frac{\text{Δ}\left(1/\text{T}\right)}{\text{Δ}\mathrm{lg}\left(1-{\text{α}}_{i}\right)}\;$. Therefore, the degradation activation energy (*E*_*i*_) can be calculated from the slope. In the absence of DTBP, the degradation activation energy *E_0_* is 2.37 eV. In the presence of DTBP, the degradation activation energies corresponding to the first and second weight loss peaks are *E*_*1*_ = 1.36 eV and *E*_*2*_ = 1.97 eV, respectively. It is clear that DTBP decreases the activation energy of PAMS. The decrease of activation energy can effectively initiate the thermolysis and make the depolymerization reaction occur more easily. Actually, the degradation of PAMS is a complex macromolecular reaction including fast and slow processes. Previous works show that depolymerization and hydrogen-transfer-induced chain scission are the two main processes during the degradation of PAMS ^19^. However, the addition of DTBP greatly changes the PAMS thermolysis mechanism. Though studies have confirmed that the degradation initiation effect is owing to the hydrogen abstraction by free radicals dissociated from DTBP. However, the specific hydrogen transfer pathways are still unknown. To reveal the degradation enhancement mechanism of PAMS in the presence of DTBP, the density functional theory (DFT) method was employed to simulate the initiation pathways.

### Initiation mechanism

**Figure 4:**
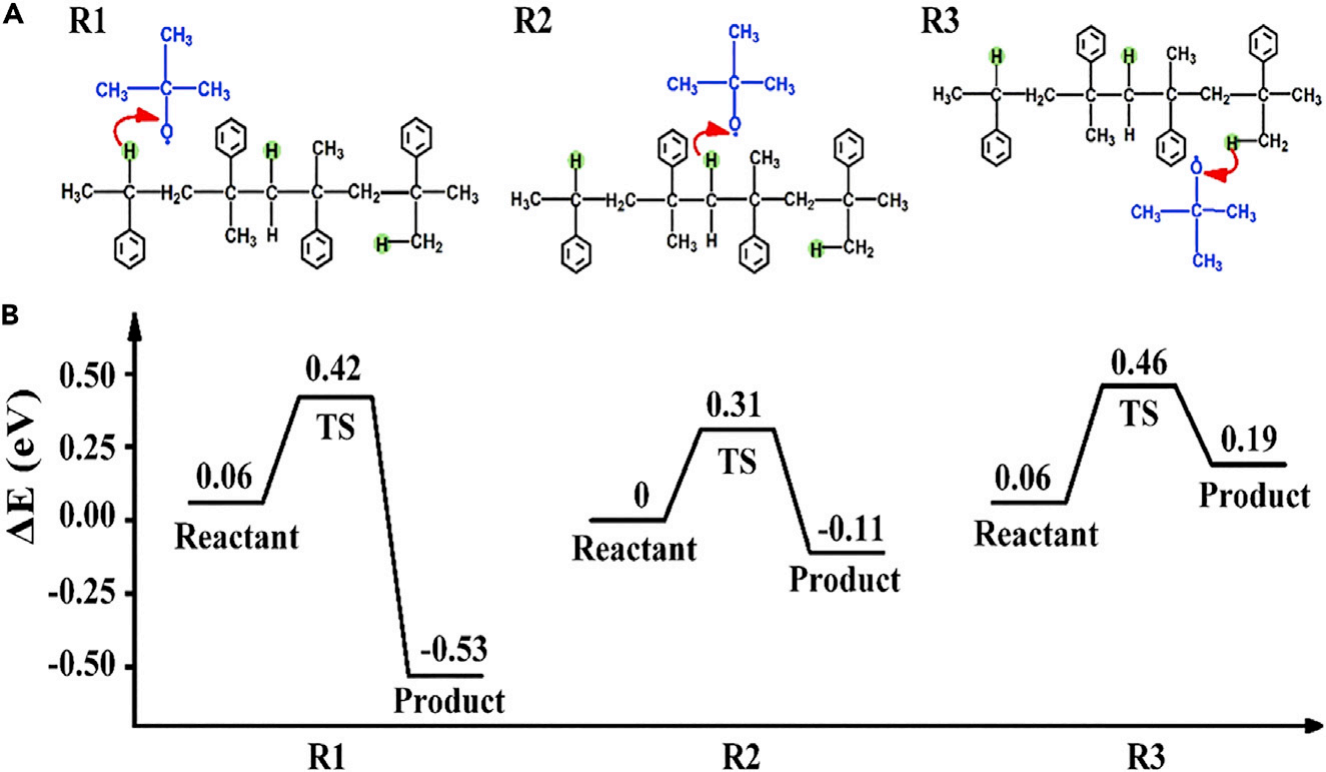
Three kinds of free radical-type reactions of seizing hydrogen atoms from saturated PAMS (A) Schematic diagram of reaction pathways. R1-R3 are reactions in which peroxide radicals seize tertiary hydrogen, secondary hydrogen, and primary hydrogen, respectively. The red arrows represent the direction in which hydrogen is seized. (B) Potential energy curves of these reactions. The energy of the reactant in R2 is taken as zero.

Various possible initiation pathways of peroxide radicals produced by the decomposition of DTBP were studied. As the electronegativity of oxygen atoms (3.44) is higher than that of carbon atoms (2.55) (Allred, 1961), peroxide radicals achieve the initiation effect by seizing the hydrogen atoms on PAMS. According to their environment and location, the hydrogen atoms can be divided into three categories, tertiary hydrogen, secondary hydrogen, and primary hydrogen; thus, three kinds of hydrogen transfer reactions are possible, named R1-R3, respectively (Figure 4A). The calculated PESs are shown in Figure 4B. The energy barriers for R1 (0.36 eV) and R2 (0.31 eV) are smaller than that for R3 (0.40 eV). More importantly, the first two reactions are exothermic, especially R1, which can reach approximately 0.60 eV, leading to substantially higher reaction rates. In contrast, R3 is a heat absorption reaction, indicating that it is relatively difficult for peroxide radicals to seize primary hydrogen. This result can be attributed to the lower chemical reaction activity of primary hydrogen compared to secondary and tertiary hydrogens (Bruice, 2010). Moreover, the energy barriers of the above three reactions are smaller than that of the depolymerization reaction (approximately 0.70 eV in Ref Zhu et al., 2022), which means that these reactions can be performed at lower temperatures. This numerical simulation results are consistent with the results shown in Figure 3.

### Dynamic simulations

**Figure 5:**
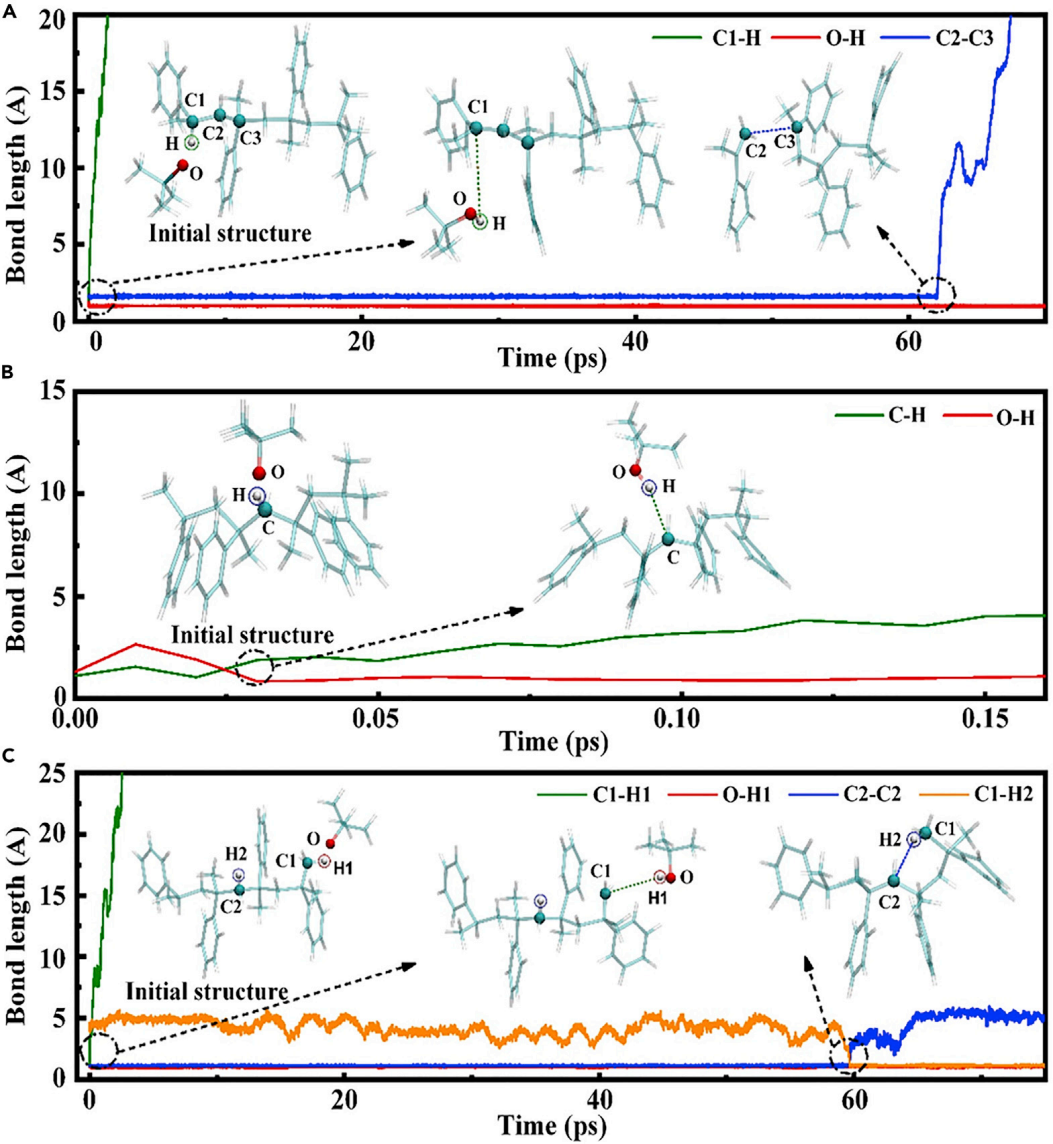
Dynamic simulations of the reaction processes of PAMS in the presence of DTBP at 550 K (A–C) These figures indicate that the length of related bonds in the reaction process changes with time when the peroxide radical is close to the (A) tertiary hydrogen, (B) secondary hydrogen, and (C) primary hydrogen of PAMS. Typical structures during reactions are also drawn. The first conformation is the initial structure.

To verify these reaction pathways of PES calculations, dynamic simulations using the DFTB-D method were further performed. As shown in Figures 5A–5C, three kinds of hydrogen transfer reactions were observed from the change in the bond length with time at the position of the related bonds, and results consistent with the PES calculations were presented. Specifically, when primary hydrogen is seized, it leads to the formation of primary carbon radicals. At this point, because of the high activity of this radical, it will further seize the hydrogen on the secondary carbon of the main chain. Afterward, a structure containing secondary carbon radicals is formed (Figure 5C). Interestingly, the formation of this structure is similar to the case of transfer for secondary hydrogen (Figure 5B). Very importantly, we found that the transfer of tertiary hydrogen located at the head end will lead to the generation of head-end unsaturated PAMS, which further depolymerizes to produce monomers (Figure 5A). This result agrees with the formation process of monomers that we have studied previously (Yu et al., 2015). Moreover, these findings further illustrate that depolymerization is a common process regardless of the presence or absence of DTBP in PAMS.

### Morphologies of the inner surface of glow discharge polymer target after mandrel degradation

**Figure 6:**
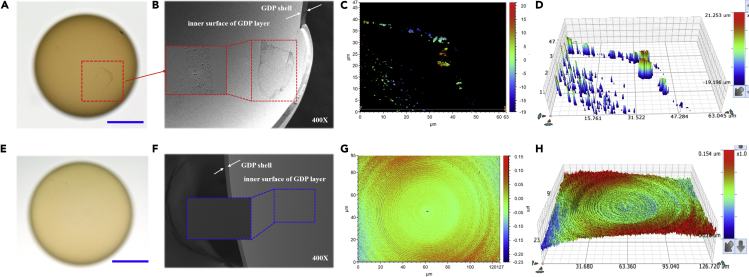
Morphologies of the GDP targets after mandrel degradation (A–D) The morphologies of the GDP targets after mandrel degradation in the absence of DTBP: (A) optical microphotograph of GDP target, (B) SEM micrographs of the residues onside the inner surface of GDP shell, (C) 2D WLI micrograph of the residues onside the inner surface of GDP shell, (D) 3D WLI micrograph of the residues onside the inner surface of GDP shell. (E–H) The morphologies of the GDP targets after mandrel degradation in the presence of DTBP: (E) optical microphotograph of GDP target, (F) SEM micrographs of the inner surface of GDP shell, (G) 2D WLI micrograph of the inner surface of GDP shell, (H) 3D WLI micrograph of the inner surface of GDP shell. The scale bars in optical microphotographs are 500 μm. The magnification of SEM micrographs is 400.

To discuss the effects of the enhanced thermal degradation of PAMS on the inner surface finish of the GDP target, the morphologies of the inner surface of GDP targets were characterized. In the absence of DTBP, there are degradation residues (Figure 6A) onside the inner surface of the GDP target after mandrel degradation. From the SEM micrograph shown in Figure 6B, the residue is hollow-ring structure and consists of lots of small particles. The 2D and 3D WLI images shown in Figures 6C and 6D also illustrate the hollow-ring structure of the degradation residue. In contrast, in the presence of DTBP, the inner surface of the GDP target after mandrel degradation is smooth and the degradation residue is obviously decreased (Figures 6A and 6B). From the 2D and 3D WLI images (Figures 6C and 6D), the inner surface is uniform after mandrel degradation. These results indicate that the enhanced thermal degradation of PAMS microspheres can greatly improve the inner surface finish of the GDP target, which is significant to the ICF implosion performance.

### Conclusions

In summary, we have demonstrated the feasibility of controlled preparation of PAMS microspheres with uniform geometry and enhanced degradation by introducing DTBP as a degradation initiator. The coefficient of variation value of the size distribution of PAMS microspheres was less than 2%, indicating the size of the PAMS microspheres was monodisperse. The effects of peroxide on PAMS degradation were experimentally and theoretically investigated in detail. TG experiments showed that the initial degradation temperature of PAMS decreases from 550 K to 450 K under the action of DTBP. DTG curves showed that there is a main weight loss peak at 571 K in the absence of DTBP. However, there were two main weight loss peaks at 484 and 602 K in the presence of DTBP. Arrhenius equation-based theoretical calculations were adopted to analyze the degradation initiation mechanism. Results showed that the degradation activity energy of PAMS decreases from 2.37 eV in the absence of DTBP to 1.36 eV in the presence of DTBP. Furthermore, the PESs of three possible hydrogen transfer were calculated and verified by the DFT method. Results showed that the energy barriers for the three reactions are 0.36, 0.31, and 0.40 eV, respectively, which are all smaller than that of the PAMS depolymerization reaction (approximately 0.70 eV). DTBP dissociates to free radicals that abstract hydrogen from the PAMS backbone to decrease the energy barriers and initiate thermal degradation. Therefore, it can be confirmed that DTBP alters the PAMS degradation mechanisms and initiate thermal degradation. The research methods in this work could provide new insight into the investigation of polymer thermal degradation. This will bring the preparation of monodisperse PAMS microspheres with enhanced thermal degradation, and thus achieve more efficient fabrication of ICF targets with improved qualities.

### Limitations of the study

This work presents a method to enhance the thermal degradation of mandrel material with di-*tert*-butyl peroxide and thus reduce the degradation residue. Therefore, the inner surface finish of the GDP target is improved. Considering that the diversity of degradation products is the main reason for degradation residues, the effects of di-*tert*-butyl peroxide on the diversity of the degradation products remain to be further explored.

## STAR★Methods

### Key resources table

**Table:** 

REAGENT or RESOURCE	SOURCE	IDENTIFIER
**Chemicals, peptides, and recombinant proteins**		

Poly-α-methylstyrene	Southwest University of Science and Technology	CAS: 25,014-31-7
Fluorobenzene	Sigma-Aldrich	CAS: 462-06-6
Di-*tert*-butyl peroxide	Aladdin Bio-Chem Technology Co., Ltd.	CAS: 110-05-4
Poly (vinyl alcohol)	Aladdin Bio-Chem Technology Co., Ltd.	CAS: 9002-89-5
Aceton	Sigma-Aldrich	CAS: 67-64-1
n-Octadecyltrimethoxysilane	Sigma-Aldrich	CAS: 3069-42-9
(3-Aminopropyl) triethoxysilane	Sigma-Aldrich	CAS: 919-30-2

**Software and algorithms**		

Q-Chem 5.4	https://www.q-chem.com/	N/A
DFTB+19.1	https://dftbplus.org/about-dftb/	N/A

### Resource availability

#### Lead contact

Further information and requests for resources should be directed to and will be fulfilled by the Lead Contact, Z.G. Wang (wangzg@jlu.edu.cn).

#### Materials availability

This study did not generate new reagents.

### Experimental model and subject details

This study does not use experimental methods typical in the life sciences.

### Method details

#### Materials source

PAMS ($\bar{Mw}$ =280 kg·mol-1) was synthesized by Southwest University of Science and Technology and used without further treatment. Fluorobenzene (FB) purchased from Sigma-Aldrich was purified by distilling at 85°C. DTBP was purchased from Aladdin Bio-Chem Technology Co., Ltd. and used as received. Poly (vinyl alcohol) (PVA, $\bar{Mw}$ = 13–23 kg·mol-1, 87–89% hydrolyzed) was also provided by Aladdin Bio-Chem Technology Co., Ltd. The reagents used for capillary treatments including aceton, n-octadecyltrimethoxysilane (OTS) and (3-Aminopropyl) triethoxysilane (3-APTES) were purchased from Sigma-Aldrich, USA. Purified water with a specific resistance of 18.3 MΩ cm was generated from a Millipore-Q water purification device. All aqueous solutions used in this work was prepared with purified water. Glass slides and glass capillaries (with inner and outer diameters of 300 × 400 μm,600 × 840 μm and 1500 × 1800 μm) used for assembling of microfluidic chips were purchased from Sail Brand company and World Precision Instruments Co., Ltd., respectively. 5% OTS in aceton solution was prepared for hydrophobic pretreatment of glass capillaries. 5% 3-APTES in aceton solution was prepared for hydrophilic pretreatment of glass capillaries. 12% PAMS in FB solutions containing different mass fractions of DTBP (0%, 1%, 3%, 5%) were prepared and used as the middle phases of W1/O/W2 compound droplets. 2% PVA aqueous solution was prepared and used as the continuous phase of W1/O/W2 compound droplets.

#### The fabrication of PAMS microspheres with microencapsulation method

A microfluidic based microencapsulation technique was adopted for the fabrication of PAMS microspheres (details can be seen Figure S1). Firstly, a co-flow microfluidic chip was assembled by coaxially aligning three cylindrical capillaries to prepare the W1/O/W2 compound droplets, where the middle capillary was hydrophobic pretreated with OTS solution, the inner and outer glass capillaries were hydrophilic pretreated with 3-ATPES solution. In the present experiment, the aforementioned deionized water, 12% PAMS/FB solution and 2% PVA aqueous solution were used as the inner, middle and outer phases, respectively. The three phases were separately delivered into the co-flow microfluidic chip by three syringe pumps (PHD ULTRA Advanced Syringe Pumps, Harvard Apparatus, Inc.), respectively. The typical flow rates of the inner, middle and outer phases were 1 mL h^−1^, 1 mL h^−1^ and 200 mL h^−1^, respectively. The monodisperse W1/O/W2 compound droplets were then continuously generated. The inner (*d*_*di*_) and outer diameters (*d*_*do*_) of the compound droplets in this work are about 950 and 1350 μm, respectively (details can be seen Figure S2 (a)). The coefficient of variation ($CV=100\%\times {\left(\sum_{i}^{n}\frac{{\left(d_{i}-\bar{d}\right)}^{2}}{n-1}\right)}^{\frac{1}{2}}/\bar{d}$) values of the size of inner and outer droplets are 1.5% and 1.75%, respectively. All CV values are less than 2%, indicating the size distributions are monodisperse.

The generated W1/O/W2 compound droplets were then collected in a flask partially filled with PVA solution for solidification. The flask was then transferred to a thermostatic water bath with a temperature of 25°C and gently rotated at 25 rpm. During the solidification, the FB molecules in the O phase continuously diffused into the outer water phase and the liquid droplets generally transferred to the solid-shelled PAMS microspheres encapsulating inner water drops. The inner drops were then removed in a vacuum oven at 45°C and the hollow core PAMS microspheres were harvested. As illustrated in Figure S2 (b), the inner (*d*_*si*_) and outer diameters (*d*_*so*_) of the solidified PAMS microspheres are 925 and 1100 μm, respectively.

#### Characterization

The optical microphotographs of the W1/O/W2 compound droplets and PAMS microspheres were obtained by a digital microscopy (VXH Keyence, Japan). The core-sheath structure was characterized with an X-ray radiography. The outer surface of the PAMS microspheres was characterized by a WLI microscopy (WYKD-NT1100) in the phase-shift interferometry (PSI) mode and SEM

The degradation performance of PAMS microspheres was analyzed by TG. The instrument used for TG analysis was a TGA instrument (Perkin Elmer, USA). The loading amount of the fractured PAMS microsphere used for TG analysis was 3–5 mg. The microspheres sample was heated from 50°C to 600°C at a scanning rate of 10 °C/min under nitrogen atmosphere.

#### Theoretical calculation methods

The effects of DTBP on PAMS degradation performance were also theoretically investigated. In this part, the PESs of various possible initiation pathways were studied based on DFT. Specifically, the extreme points on the PESs were fully optimized using the hybrid exchange-correlation functional Becke-3-Lee-Yang-Parr with empirical dispersion correction (B3LYP-D3) (Becke, 1993; Lee et al., 1988), which was appropriate for describing the geometric and electronic properties of organic molecules. A split-valence double-zeta plus polarization basis set was used for C, H and O atoms. Then, vibrational frequency calculations were performed at the same level to ensure the stability of the optimized structures. The above calculations were implemented in the version 5.4 release of the Q-Chem package (Epifanovsky et al., 2021). In dynamics simulations, the density functional tight-binding method with empirical dispersion correction (DFTB-D) was adopted (Elstner et al., 1998). Under the requirement of the maximum accuracy, the calculation speed of this method is 10^3^- to 10^4^-fold faster than that of the DFT calculation; thus, it has been successfully applied to study various macromolecules. The Slater–Koster-type parameters were chosen to describe the connections between atoms in the system. The geometric structure of a system containing elements such as C, H and O described by the DFTB method based on these parameters was consistent with that described by the traditional DFT methods (Krüger et al., 2005). The dynamic simulations were performed by the DFTB+ 19.1 program (Hourahine et al., 2020).

### Quantification and statistical analysis

Analyses and plots were performed with Microsoft Excel, PowerPoint and MATLAB.

## Data Availability

Data - Data reported in this paper will be shared by the lead contact upon request. Code - No new code was generated during the course of this study. Other - Any additional information required to reanalyze the data reported in this paper is available from the lead contact upon request.

## Supplemental information

DocumentS1. Figures S1 and S2
