# Supplementary material for: Initiator enhancement of mandrel degradation for ICF target fabrication
Source: iScience. 2022 Jul 9;25(8):104733. doi: 10.1016/j.isci.2022.104733 (PMC9307930; doi:10.1016/j.isci.2022.104733)
Supplement: DocumentS1. Figures S1 and S2 [file mmc1.pdf]

iScience, Volume *25*

Supplemental information

Initiator enhancement of mandrel degradation for ICF target fabrication

Qiang Chen, Yu Zhu, Zhanwen Zhang, Jiajun Ma, Zhibing He, and Zhigang Wang

Figure S1. Schematic of the fabrication of PAMS microspheres with microencapsulation method, Related to STAR Methods

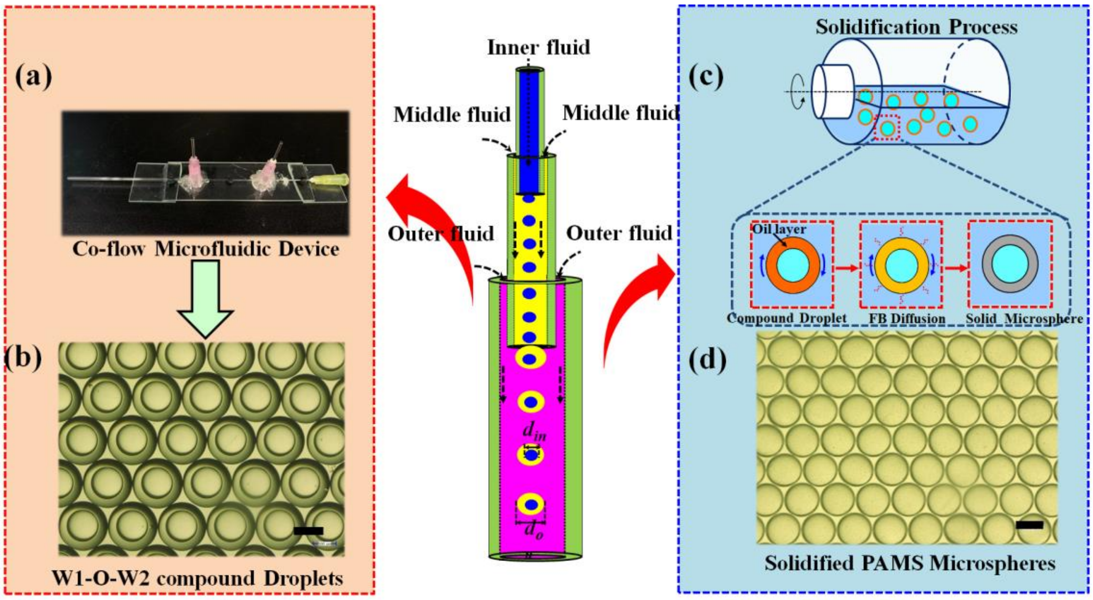

Schematic of the fabrication of PAMS microspheres with microencapsulation method. (a) Co-flow microfluidic chip. (b) Optical microphotograph of the W1/O/W2 compound droplets. (c) Solvent evaporation-based solidification of W1/O/W2 compound droplets. (d) Optical microphotograph of the solidified PAMS microspheres.

Figure S2. The size distributions of the W1/O/W2 compound droplets and the solidified PAMS microspheres, Related to STAR Methods

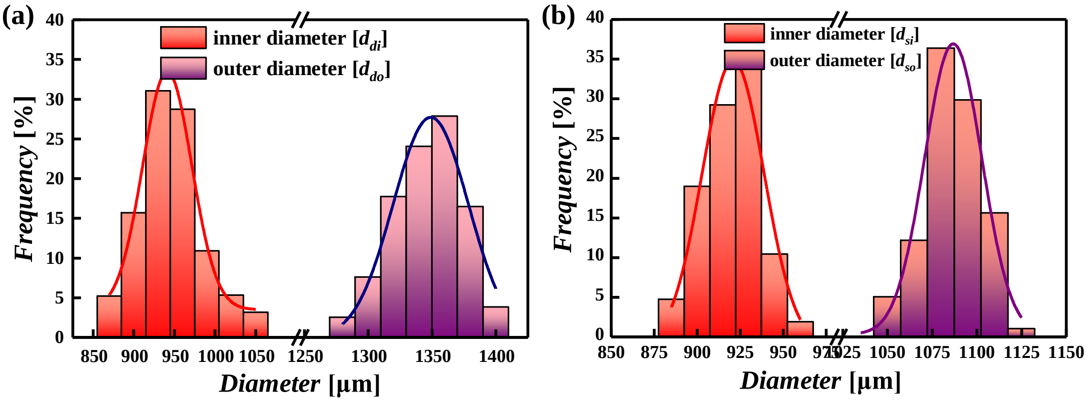

The size distributions of the W1/O/W2 compound droplets and the solidified PAMS microspheres. (a) The inner (d_{di}) and outer diameters (d_{do}) of the W1/O/W2 compound droplets. (b) The inner (d_{si}) and outer diameters (d_{so}) of the PAMS microspheres.
